# Time to Decompression in Obstructive Urosepsis from Ureteral Calculi: Thresholds, Initial Diversion, and Early Biomarkers: A Systematic Review

**DOI:** 10.3390/jcm14238546

**Published:** 2025-12-02

**Authors:** Adela Benea, Daniel Porav-Hodade, Mirela Turaiche, Ovidiu Rosca, Daniel-Florin Lighezan, Ciprian Rachieru, Livia Stanga, Adrian Cosmin Ilie, Oana Silvana Sarau, Cristian Andrei Sarau

**Affiliations:** 1 Doctoral School, Faculty of Medicine, “Victor Babes” University of Medicine and Pharmacy Timisoara, 300041 Timisoara, Romania; adela.benea@umft.ro; 2 Methodological and Infectious Diseases Research Center, Faculty of Medicine, “Victor Babes” University of Medicine and Pharmacy Timisoara, 300041 Timisoara, Romania; paliu.mirela@umft.ro (M.T.); rosca.ovidiu@umft.ro (O.R.); 3 Department of Urology, The “George Emil Palade” University of Medicine, Pharmacy, Science, and Technology of Targu Mures, 540139 Targu Mures, Romania; daniel.porav-hodade@umfst.ro; 4 Department I of Internal Medicine, Faculty of Medicine, “Victor Babes” University of Medicine and Pharmacy Timisoara, 300041 Timisoara, Romania; dlighezan@umft.ro (D.-F.L.); ciprian.rachieru@umft.ro (C.R.); csarau@umft.ro (C.A.S.); 5 Center for Advanced Research in Cardiovascular Pathology and Hemostaseology, “Victor Babes” University of Medicine and Pharmacy Timisoara, 300041 Timisoara, Romania; 6 Discipline of Microbiology, Faculty of Medicine, “Victor Babes” University of Medicine and Pharmacy Timisoara, 300041 Timisoara, Romania; 7 Department of Functional Sciences, Discipline of Public Health, Center for Translational Research and Systems Medicine, “Victor Babes” University of Medicine and Pharmacy Timisoara, 300041 Timisoara, Romania; 8 Department V of Internal Medicine, Discipline of Hematology, “Victor Babes” University of Medicine and Pharmacy Timisoara, 300041 Timisoara, Romania; oana.sarau@umft.ro

**Keywords:** ureteral calculi, sepsis, pyelonephritis, nephrostomy, percutaneous, procalcitonin

## Abstract

**Background/Objectives**: Obstructive urosepsis mandates rapid source control, yet actionable time-to-decompression (TTD) thresholds and the influence of diversion route remain debated. This review synthesized evidence on TTD, early outcomes, and predictive biomarkers. **Methods**: Following PRISMA-2020, ten studies met eligibility: three large administrative cohorts and seven clinical series/trials comparing outcomes by TTD and/or initial decompression (retrograde stent [RUS] vs. percutaneous nephrostomy [PCN]) and reporting biomarkers. **Results**: Delays were consistently harmful. In a national cohort, a TTD of ≥ 2 days increased in-hospital mortality (adjusted OR, 1.29; 95% CI, 1.03–1.63). Another analysis showed mortality of 0.16% with ≤48 h vs. 0.47% when delayed (derived OR, 0.43; *p* = 0.044). Absence of decompression yielded the highest mortality (19.2% vs. 8.82%; OR, 2.6; 95% CI, 1.9–3.7). Septic shock remained frequent despite low crude mortality in clinical series: 20.8% and 33.3% across two cohorts. Post-decompression urosepsis occurred in 18.7% in a randomized study and was associated with pyonephrosis and higher procalcitonin (PCT). An ED cohort showed that earlier stenting reduced length of stay (≤6 h: 35.6 h vs. 71.6 h, *p* = 0.01; ≤10 h: 45.7 h vs. 82.4 h, *p* = 0.04). Modality effects were modest; one cohort reported higher ICU use with PCN vs. RUS (OR, 3.23; 95% CI, 1.24–8.41), likely reflecting confounding by indication. **Conclusions**: Across designs, timeliness—not device choice—was the dominant determinant of early outcomes. Biomarkers (notably, PCT) and imaging features can prioritize ultra-early decompression.

## 1. Introduction

Obstructive urosepsis from ureteral calculi is a time-critical emergency in which source control by urinary drainage must be secured without delay; contemporary guideline syntheses consistently emphasize urgent decompression in any infected, obstructed system [1]. From a sepsis-system standpoint, timeliness is not cosmetic: the Surviving Sepsis Campaign recommends achieving source control as soon as is feasible—ideally within hours—because every increment of delay compounds organ dysfunction and mortality [2]. Multispecialty analyses across heterogeneous sources of infection quantify this relationship, showing higher 90-day mortality when source control is deferred beyond early thresholds (for example, after approximately six hours in community-acquired sepsis) [3]. Within urolithiasis populations, large administrative cohorts underscore the clinical burden of stone-associated infection and sepsis, which disproportionately affect women and those with prior urinary infections, reinforcing why stones plus infection should trigger standardized, time-bound pathways to decompression [4].

Debate persists about the initial decompression modality—retrograde ureteral stenting (RUS) versus percutaneous nephrostomy (PCN)—but the most recent systematic review and meta-analysis in infected, obstructed urolithiasis found broadly comparable early efficacy (defervescence, leukocyte normalization, hospital stay, and technical success), suggesting that logistics and availability often determine the “right” procedure in the first hours [5]. Narrative and guideline-overview reviews reach similar conclusions: both PCN and RUS are valid, and peri-procedural risks and downstream quality-of-life trade-offs should be balanced against the imperative of timely source control [6]. Beyond the index hospitalization, meta-analytic signals indicate that patient-level and anatomic factors (e.g., stone size/burden and hydronephrosis grade) shape recovery trajectories and re-intervention risks more than the initial drainage route per se [7]. These observations collectively imply that when we decompress may be a stronger early determinant of survival than how we decompress.

Emerging work also clarifies how in-hospital processes influence deterioration while patients wait for drainage. In a national analysis of obstructing-stone admissions with suspected infection, the interval from presentation to decompression and the timing of subsequent definitive ureteroscopy did not increase post-procedural urosepsis when initial source control was achieved promptly—supporting a strategy that prioritizes rapid drainage first and definitive stone treatment later [8]. Parallel prognostic research has focused on bedside biomarkers to identify patients most likely to decompensate without very early drainage. In obstructive acute pyelonephritis associated with ureteral stones (OAPN-US), a machine-learning–assisted model combining procalcitonin (PCT), the absolute lymphocyte count, and INR accurately predicted septic shock, with PCT contributing the greatest discriminative weight [9]. A complementary nomogram in urolithiasis-related obstructive pyelonephritis (UROP) flagged diabetes, a higher shock index, an elevated neutrophil-to-lymphocyte ratio (NLR), and raised CRP as early sepsis predictors [10], while emergency-department NLR itself has been associated with the need for urgent stenting in stone-related infection [11].

Independent of PCT, thrombocytopenia and hypoalbuminemia repeatedly correlate with septic shock in obstructive pyelonephritis, helping clinicians recognize patients whose physiologic reserve is already collapsing [12]. Authoritative clinical references likewise codify that obstructive pyelonephritis/pyonephrosis constitutes a surgical emergency: deferring drainage risks rapid bacteremic progression and death, and comprehensive evaluation should proceed in parallel with decompression rather than in sequence [13]. Imaging can also refine triage in real time. Quantitative CT characteristics (e.g., stone and collecting-system Hounsfield attenuation) distinguish pyonephrosis from sterile hydronephrosis and align with pus burden at drainage [14]; newer radiomics-based models integrate CT texture features with clinical parameters to anticipate pyonephrosis pre-operatively, potentially shortening the path to the operating room for the highest-risk patients [15].

This systematic review aims to (i) delineate clinically actionable time-to-decompression (TTD) thresholds in obstructive urosepsis from ureteral calculi and their associations with mortality, septic shock, ICU admission, and length of stay; (ii) compare initial decompression modalities (PCN vs. RUS) insofar as they modify early outcomes after accounting for TTD; and (iii) synthesize evidence on early biomarkers/labs (particularly PCT, platelets, albumin, NLR, and shock index) that stratify deterioration risk before drainage. We hypothesize that TTD within a 24–48 h window (and likely within hours for the sickest) is independently associated with lower short-term mortality and shock, that modality has a smaller effect size than timeliness on these early outcomes, and that biomarker-guided triage can identify a subgroup needing near-immediate decompression. The novelty of this work is a TTD-centered framework that integrates thresholds, modality-agnostic logistics, and biomarker-based prioritization to support measurable quality metrics and operational redesign in emergency stone sepsis pathways.

## 2. Materials and Methods

### 2.1. Protocol, PICO Statement, and Eligibility Criteria

This review adhered to PRISMA-2020 [16] (Supplementary Materials) and followed a prospectively specified protocol (OSF registration ID osf.io/hta5d). Objectives, eligibility criteria, outcomes, and analytic plans were locked a priori; no post hoc changes affecting study selection or synthesis were introduced.

PICO statement: this study aimed to determine whether, in adults with infection due to ureteral calculi causing upper-tract obstruction, earlier urinary decompression (retrograde ureteral stenting or percutaneous nephrostomy) versus later/no decompression improves short-term mortality, septic shock, ICU use, and length of stay and which pre-drainage biomarkers stratify deterioration risk.

The current study included randomized trials and comparative observational studies enrolling adults (≥18 years) with infected, obstructed ureteral stones that reported time to decompression (TTD) and/or initial decompression modality with at least one prespecified clinical outcome. Mixed-etiology obstruction cohorts were eligible if calculi + infection data were extractable or comprised ≥70% of the sample. In practice, all included analyses either exclusively enrolled obstructive uropathy due to ureteral/upper-tract calculi or reported results in which calculous obstruction constituted the clear majority of cases; we did not pool outcomes from purely malignant or non-calculous obstruction with stone-related urosepsis.

### 2.2. Information Sources and Search Dates

Three databases—PubMed/MEDLINE, Embase (Elsevier), and Scopus (Elsevier)—were searched from inception to 10 September 2025. Reference lists of included studies and pertinent reviews were hand-searched. The search strings included the following:

PubMed/MEDLINE: (“Ureteral Calculi” [Mesh] OR “Urinary Calculi” [Mesh] OR urolithiasis [tiab] OR “ureteral calculi” [tiab] OR “ureteral stone*” [tiab] OR “ureteric stone*” [tiab] OR “upper urinary tract stone*” [tiab]) AND (“Sepsis” [Mesh] OR “Shock, Septic” [Mesh] OR sepsis [tiab] OR “septic shock” [tiab] OR “urinary tract infection*” [tiab] OR “pyelonephritis” [tiab] OR “obstructive pyelonephritis” [tiab] OR pyonephrosis [tiab]) AND (“Ureteral Obstruction” [Mesh] OR obstruct* [tiab] OR hydronephrosis [tiab]) AND (decompression [tiab] OR drainage [tiab] OR “source control” [tiab] OR “percutaneous nephrostom*” [tiab] OR nephrostom* [tiab] OR “ureteral stent*” [tiab] OR stent* [tiab]) AND (time* [tiab] OR delay* [tiab] OR “time-to” [tiab] OR timing [tiab] OR early [tiab] OR urgent [tiab] OR emergen* [tiab]) NOT (animals [mh] NOT humans [mh]).

Embase (Elsevier; Emtree mapped): (‘ureter calculus’/exp OR ‘urinary calculus’/exp OR urolithiasis:ti,ab OR ‘ureteral calculi’:ti,ab OR ‘ureteral stone*’:ti,ab OR ‘ureteric stone*’:ti,ab OR ‘upper urinary tract stone*’:ti,ab) AND (‘sepsis’/exp OR ‘septic shock’/exp OR sepsis:ti,ab OR ‘septic shock’:ti,ab OR ‘urinary tract infection’/exp OR ‘urinary tract infection*’:ti,ab OR ‘pyelonephritis’/exp OR pyelonephritis:ti,ab OR pyonephrosis:ti,ab) AND (‘ureter obstruction’/exp OR obstruction:ti,ab OR obstructive:ti,ab OR hydronephrosis:ti,ab) AND (decompression:ti,ab OR drainage:ti,ab OR ‘source control’:ti,ab OR ‘percutaneous nephrostomy’/exp OR ‘percutaneous nephrostom*’:ti,ab OR nephrostom*:ti,ab OR ‘ureteral stent’/exp OR ‘ureteral stent*’:ti,ab OR stent*:ti,ab) AND (time*:ti,ab OR delay*:ti,ab OR ‘time to’:ti,ab OR timing:ti,ab OR early:ti,ab OR urgent:ti,ab OR emergen*:ti,ab) NOT ([animals]/lim NOT [humans]/lim).

Scopus (Elsevier; TITLE-ABS-KEY): TITLE-ABS-KEY ((“ureteral calculi” OR “ureteral stone*” OR “ureteric stone*” OR urolithiasis OR “upper urinary tract stone*”) AND (sepsis OR “septic shock” OR “urinary tract infection*” OR pyelonephritis OR pyonephrosis) AND (obstruct* OR hydronephrosis) AND (decompression OR drainage OR “source control” OR “percutaneous nephrostom*” OR nephrostom* OR “ureteral stent*” OR stent*) AND (time* OR delay* OR “time-to” OR timing OR early OR urgent OR emergen*)).

### 2.3. Study Selection and PRISMA Flow

**Figure 1:**
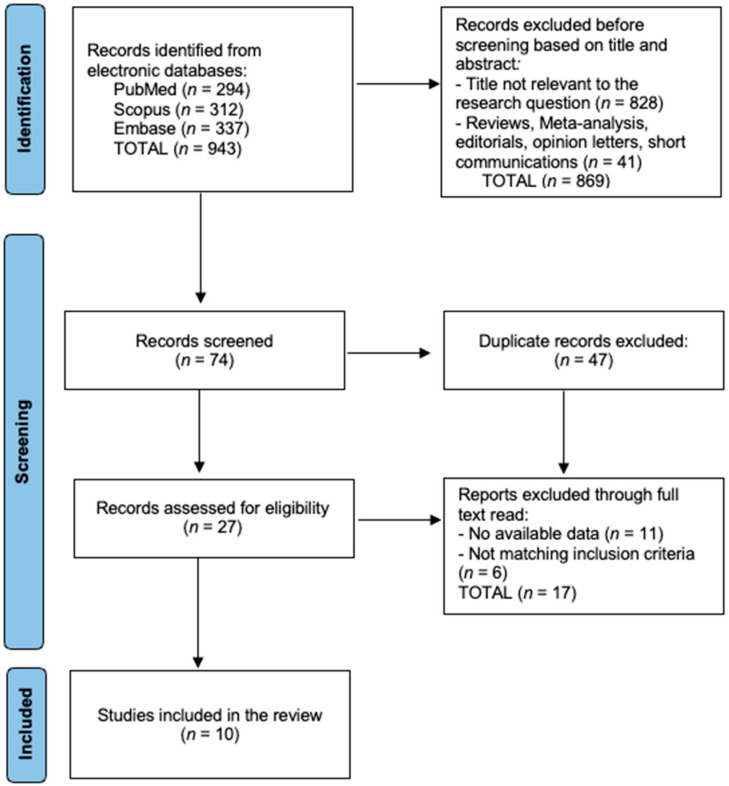
PRISMA flowchart.

Records were exported to EndNote X20 for de-duplication and screened in Rayyan QCRI by two independent reviewers (calibration κ ≥ 0.80). The PRISMA flow mirrored Figure 1: a total of 943 records were identified from the databases (PubMed = 294; Scopus = 312; Embase = 337). Before formal screening, 869 records were excluded at the title/abstract stage for irrelevance (n = 828) or article type (reviews/meta-analyses/editorials/short communications, n = 41). Seventy-four records entered screening; a total of 47 duplicates were removed, leaving 27 full texts assessed for eligibility. Seventeen full texts were excluded—there were no available/usable data (n = 11), or the inclusion criteria were not met (n = 6). Ten studies met all criteria and were included in the review.

### 2.4. Data Items and Extraction

We extracted the study design/setting; eligibility and case definitions (obstruction and infection/sepsis criteria); stone level/size; exposure definitions (TTD—continuous and thresholds; initial PCN vs. RUS); co-interventions; and outcomes (in-hospital/30-day mortality, septic shock/vasopressors, ICU/organ support, length of stay, technical success/failure, complications, readmission, and time to definitive ureteroscopy). Biomarkers (procalcitonin, CRP, NLR, platelets, and albumin), urine/blood culture results, and imaging indicators (hydronephrosis grade and pyonephrosis) were abstracted when available.

TTD was the interval from presentation (ED or admission) to first effective drainage. Mortality was harmonized to in-hospital or 30-day mortality. ICU escalation included admission and/or organ support. When necessary, medians (IQRs) were converted to means (SDs) using standard methods, and time units were standardized (hours/days). Biomarkers were harmonized to conventional units (PCT ng/mL). Given the limited number of TTD studies per outcome and substantial heterogeneity in exposure definitions (continuous vs. categorical TTD, different time cut-offs, and presence vs. absence of decompression), sepsis criteria, and case mix, we prespecified a narrative synthesis without a formal meta-analysis. Effect directions and magnitudes were summarized descriptively and graphically rather than pooled in a random-effects model.

### 2.5. Risk-of-Bias Assessment

Two reviewers independently assessed the risk of bias using RoB 2 (randomized trials) and ROBINS-I (non-randomized studies), prespecifying confounders (baseline severity, comorbidity, bacteremia, pyonephrosis, stone level/size, weekend presentation, and concurrent process changes). QUIPS/QUADAS-2 domains were referenced for prognostic/diagnostic elements where applicable. Disagreements were resolved by consensus. Risk of bias assessment of included studies [17,18,19,20,21,22,23,24,25,26] is presented in Table 1.

## 3. Results

Across ten primary studies [17,18,19,20,21,22,23,24,25,26], three analyses used large U.S. administrative datasets (Haas, n = 311,100; Blackwell, n = 10,301; Borofsky, n = 1712) to evaluate the relationship between decompression timing and mortality in obstructing-stone infection [17,18,19]. Four single-center cohorts from Japan, the U.S., and China assessed septic shock risk and care processes in obstructive acute pyelonephritis (OAPN) requiring urgent drainage—Yamamoto (n = 98; 101 drainage events), Tambo (n = 69), Goldsmith (n = 130), and Xu (n = 110) [20,21,24,25]. Two interventional studies compared initial decompression modality: a prospective RCT in Germany (Mokhmalji, n = 40) and a prospective randomized trial in China (Lu (n = 150, systemic inflammatory response syndrome (SIRS) plus ureteral stones; randomized PCN (n = 78) vs. RUS (n = 72)) [22]). Finally, an ED cohort (Faw, n = 48) examined whether very early stenting (≤6–10 h) shortened length of stay (LOS) and reduced ICU use in obstructing ureteral stones with presumed infection [26]. Objectives were convergent: quantify time-to-decompression (TTD) thresholds linked to mortality or shock [17,18,19,20,21], compare PCN vs. RUS on early outcomes [22,23,24,25], and test process-of-care impacts on LOS/ICU [26], as seen in Table 2.

Administrative cohorts consistently show harm from delayed or absent drainage: in Haas, a decompression delay of ≥2 days increased in-hospital mortality (adjusted OR [aOR], 1.29; 95% CI, 1.03–1.63) and acute kidney injury (aOR, 1.12; 95% CI, 1.03–1.22) [17]; in Blackwell, mortality was 0.16% with timely, ≤48 h, vs. 0.47% when delayed (derived OR for timeliness, 0.43; *p* = 0.044) [18]; in Borofsky, lack of decompression was associated with higher death (19.2% vs. 8.82%; OR, 2.6; 95% CI, 1.9–3.7) [19]. In clinical cohorts, mortality was low overall (Yamamoto, 2.0% [2/98]) but septic shock occurred in 20.8% of drainage events, with longer LOS for shock vs. non-shock (14 vs. 10 days; *p* = 0.008) [20]; Tambo reported 33% shock (23/69) with strong pathogen-related predictors (non-*E. coli* predominance; OR, 10.6) [21]. Post-decompression urosepsis in Lu occurred in 18.7% (28/150) and was more likely with pyonephrosis and higher procalcitonin (PCT) [22]. Goldsmith observed low mortality (0.8%) but higher ICU needs with PCN vs. stent (OR, 3.23; 95% CI, 1.24–8.41) and longer LOS after adjustment [25]. Administrative cohorts thus consistently linked delayed or absent decompression with increased mortality, while the ED cohort by Faw suggested that stenting within 6–10 h shortens hospital stay without a clear ICU signal [17,18,19,26], as seen in Table 3. A consolidated overview of TTD definitions, thresholds, and associated outcome measures is provided in Table 4.

Technical success favored PCN in older trials (Mokhmalji: PCN, 100% vs. stent, 80%; major complications, 0% vs. 11%) [23], while in the contemporary randomized study, success remained high for both (Lu: PCN, 100% vs. RUSI, 96%), with 18.7% overall post-decompression urosepsis driven by pyonephrosis and higher PCT [22]. In a 15-year single-system cohort (Goldsmith), overall diversion failure was 2.3% (3/130), with higher baseline acuity in PCN patients (APACHE II, 15 vs. 11) and adjusted increases in ICU use and LOS for PCN vs. stent [25]. Anatomic constraints influenced stent performance in urosepsis (Xu: greater stent failure risk with proximal/UPJ obstruction; overall clinical efficacy that was otherwise similar, and fever/WBC normalization comparable to PCN), again reflecting selective triage of anatomically challenging cases to PCN rather than inherent modality inferiority [24]. Microbiology and labs reflected severe infection burden: Yamamoto reported urine culture positivity of 68.3%, bacteremia of 35.6%, and a median CRP of 16.1 mg/dL, with a median onset to drainage of 3 days and shock associated with older age and higher bacteremia rates [20]; Tambo found shock associated with positive blood cultures (59% vs. 18%) and non-*E. coli* pathogens (74% vs. 33%) [21]; and in the ED cohort, 58.3% had positive urine cultures [26]. Policy-relevant operation signals include reduced odds of timely, ≤48 h, intervention on weekends (−26%) in Blackwell [18] and the mortality penalty from not decompressing in Borofsky (OR, 2.6) [19], as presented in Table 5. Importantly, PCN was preferentially used in sicker patients with higher baseline APACHE II scores and larger stones (APACHE II, 15 vs. 11; stone size, 10 vs. 7 mm), indicating substantial confounding by indication and limiting causal interpretation of the association between PCN and ICU use/LOS [25].

**Figure 2:**
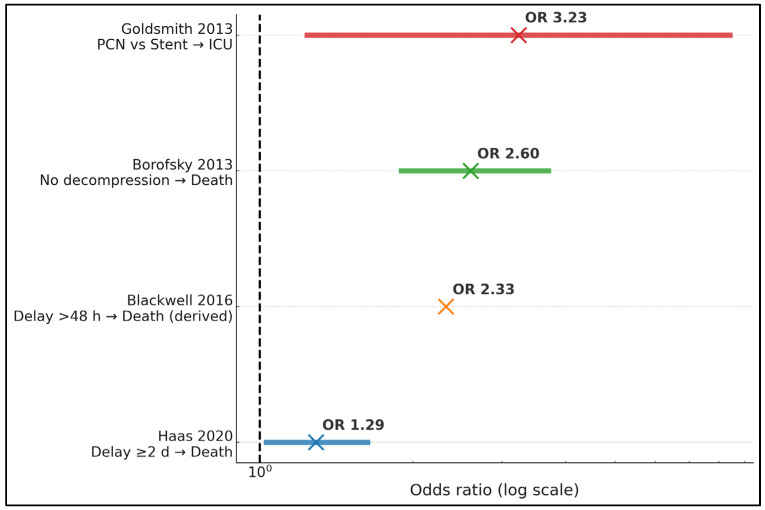
Effect sizes (odds ratios) for mortality and ICU use plotted on a log10(OR) axis [17,18,19,25]; the vertical line indicates OR = 1 (no difference). ICU—intensive care unit; OR—odds ratio; PCN—percutaneous nephrostomy.

Effect sizes aligned directionally across datasets: a delay of ≥2 days increased in-hospital death (OR, 1.29) [17]; waiting >48 h conferred a larger mortality signal (OR 2.33) [18]; absence of decompression was associated with the highest mortality among timing contrasts (OR, 2.60) [19]; and PCN was associated with greater ICU utilization than stenting (OR, 3.23) within septic obstructive urolithiasis [25]. The ascending pattern underscored consistent harm when drainage was deferred or omitted and higher escalation needs with PCN in sicker case mixes (Figure 2).

**Figure 3:**
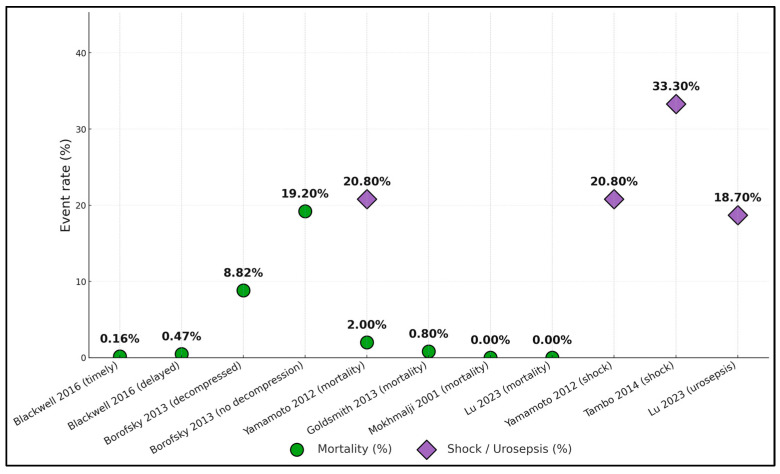
Event rates across studies [18,19,20,21,22,23,25].

Event rates clustered into a clear gradient: in administrative data, mortality was lower with timely care (0.16%) and rose with delay (0.47%) within the same cohort [18]; when decompression was not performed, death approached one in five (19.2%) versus single digits with decompression (8.82%) [19]. Contemporary clinical series and trials reported very low in-hospital mortality—2.0% [25], 0.8% [23], and 0% in two cohorts [20,22]—yet severe physiologic deterioration remained common: septic shock occurred in 20.8% [20] and 33.3% [21], and post-decompression urosepsis occurred in 18.7% [22]. Overall, the plot illustrated minimal mortality with rapid source control, a marked penalty when drainage was delayed or omitted, and substantial residual shock/urosepsis risk across settings (Figure 3).

## 4. Discussion

### 4.1. Summary of Evidence

Within the ten included studies, timeliness of source control emerged as the dominant driver of early outcomes. Administrative cohorts consistently linked delayed or absent decompression to higher in-hospital mortality and acute kidney injury [17,18,19], while single-center clinical series documented substantial rates of septic shock and post-decompression urosepsis despite low crude mortality [20,21,22]. External process-improvement studies corroborate these signals: after a hospital-wide ‘stone sepsis’ pathway was implemented, time to decompression fell substantially, and each additional hour of delay independently increased the odds of a prolonged length of stay, even after adjustment for acuity and comorbidity [27]. Regarding the initial diversion route, contemporary comparative studies outside our pool echo our finding of broadly similar short-term clinical efficacy between percutaneous nephrostomy (PCN) and retrograde ureteral stenting (RUS), with selection largely driven by anatomy and physiologic severity. This pattern is already evident within our dataset: Goldsmith [25] and Xu [24] both reported greater ICU utilization and/or longer LOS among PCN recipients, but in both studies, PCN was preferentially chosen for patients with higher APACHE II scores, larger or more proximal stones, or more complex anatomy, making these associations highly susceptible to confounding by indication rather than reflecting intrinsic modality-related harm. A separate implementation series likewise reported that protocolized triage shortened time to decompression and reduced hospitalization metrics in routine practice, reinforcing that the quality target is the interval to effective drainage rather than a specific device [28]. These operational data align tightly with the mortality penalties observed when decompression is delayed or omitted in our included cohorts.

Regarding the initial diversion route, contemporary comparative studies outside our pool echo our finding of broadly similar short-term clinical efficacy between percutaneous nephrostomy (PCN) and retrograde ureteral stenting (RUS), with selection largely driven by anatomy and physiologic severity. In a 1500-patient retrospective series of acute obstructive uropathy, Cozma et al. found no clinically important differences in fever or leukocytosis normalization between PCN and stent, while sicker or anatomically challenging cases were preferentially triaged to PCN—illustrating confounding by indication rather than intrinsic modality superiority [29]. Another cohort focusing on complication profiles reported higher technical success with PCN and fewer early complications in difficult anatomy but again emphasized that patient factors and logistics dominate the first-hours decision [30]. Taken together, these data support a modality-agnostic, logistics-first approach: choose the route that can be executed fastest and most reliably in the local setting.

Early biomarkers meaningfully stratify deterioration risk while patients await drainage. In stone-associated sepsis, an emergency-department procalcitonin (PCT) threshold of around 0.5 ng/mL predicted progression to septic shock and outperformed conventional hematologic indicators [31]. Extending this, Tambo et al. showed that both presepsin and PCT measured before intervention independently predicted Sepsis-3 sepsis in obstructive acute pyelonephritis, offering a pragmatic bedside risk tool for prioritizing near-immediate decompression [32]. These signals dovetail with our review’s observations and suggest a triage bundle in which elevated PCT (±presepsin), plus simple labs (platelet count and albumin), flags patients whose physiologic reserve is collapsing and who require urgent source control [33,34,35,36].

Imaging can further compress the pathway by anticipating pus burden and technical complexity. Two recent models combined CT radiomics (one with a 3D-CNN) and clinical features to identify calculous pyonephrosis pre-operatively with high discrimination, proposing earlier operating-room prioritization and more informed device selection when access is constrained [15,37]. Although external validation and workflow integration are still needed, these studies point to a feasible “imaging-assisted triage” strategy that can sit alongside biomarker-based risk scores, consistent with consensus imaging guidance that emphasizes CT to evaluate complicated presentations and suspected obstruction in the setting of APN [38].

Patient-level risk factors observed across the broader APN/urosepsis literature also mirror the profiles seen in our included studies. Advanced age, chronic kidney disease, and markedly elevated creatinine independently predicted progression from obstructive urosepsis to severe sepsis or septic shock in a dedicated urolithiasis cohort [36]. In obstructive urolithiasis, thrombocytopenia and hypoalbuminemia were specifically associated with septic shock, underscoring how routine labs encode severity beyond vitals alone [35]. These features align with our biomarker-and-imaging discussion and offer a practical way to “risk-load” the queue when operative capacity is limited: older patients with CKD, bacteremia, high inflammatory indices, and abnormal PCT/presepsin should move to the front of the line for decompression.

Finally, the post-drainage arc requires deliberate planning. A prolonged pre-ureteroscopy stent dwell time increases infectious complications; a multicenter European analysis identified a sharp rise in post-ureteroscopy febrile UTI when stents remained in situ beyond two months [36]. Among patients specifically decompressed for obstructive pyelonephritis, limiting pre-operative dwell to ≤21 days and avoiding long operative times reduced febrile UTI after definitive ureteroscopy [34]. These observations resonate with our process-of-care synthesis: (i) standardize rapid recognition and decompression [27,28]; (ii) select PCN or RUS based on speed and reliability in context [29,30]; and (iii) use biomarker-imaging-clinical signals to prioritize the sickest patients and to schedule definitive stone treatment within safe dwell-time windows [31,32,33,34,35,36,37,38].

For implications in clinical practice, adopt a “time first” pathway: target decompression within 24–48 h for all infected obstructions—and within hours for high-risk patients. Use whichever diversion (RUS or PCN) can be delivered fastest and most reliably in the local context. Integrate triage triggers (PCT elevation, pyonephrosis on imaging, thrombocytopenia, hypoalbuminemia, and shock index) to move the sickest to the front of the queue. After stabilization, schedule definitive stone management promptly to minimize infectious sequelae.

### 4.2. Limitations

Evidence is dominated by observational cohorts and administrative databases prone to misclassification, residual confounding (including confounding by indication for PCN), and heterogeneous TTD definitions and sepsis criteria. Sepsis was defined using SIRS-based criteria, older consensus definitions, Sepsis-3, or ICD coding, depending on the data source, and TTD was variably measured from symptom onset, emergency department triage, hospital admission, or inter-hospital transfer to effective drainage. These clinical and methodological differences, combined with the small number of studies per outcome, precluded a robust random-effects meta-analysis and led us to favor a structured narrative synthesis and graphical presentation rather than pooled estimates. Biomarker thresholds varied, and individual-patient data meta-analysis was not feasible. Some reports provided limited adjustment for illness severity, and modality comparisons were influenced by anatomy and baseline risk; accordingly, we interpreted modality-related associations (e.g., higher ICU use with PCN) as hypothesis-generating and gave greater inferential weight to the more consistent timing signals from lower-risk analyses.

## 5. Conclusions

For obstructive urosepsis due to ureteral calculi, every delay in decompression worsens outcomes; the absence of drainage is associated with the highest mortality. When capacity is constrained, prioritize speed over modality, guided by bedside risk markers (e.g., PCT) and imaging signs of pus burden. System-level protocols that guarantee rapid access to either RUS or PCN are likely to yield the greatest survival and efficiency gains.

## Figures and Tables

**Table 1:** Summary of risk-of-bias judgments for included studies.

Study (Year)	Design	Tool	Overall RoB Judgment	Main Concerns
Haas 2020 [17]	Retrospective administrative cohort	ROBINS-I	Moderate	Residual confounding (limited clinical severity data), reliance on ICD coding for exposure, and outcome definitions
Blackwell 2016 [18]	Retrospective administrative cohort	ROBINS-I	Moderate	Residual confounding, possible misclassification of infection and TTD, and “weekend effect” not fully explained by measured covariates
Borofsky 2013 [19]	Retrospective administrative cohort	ROBINS-I	Moderate	Unmeasured confounding, coding-based identification of sepsis and decompression, and limited clinical granularity
Yamamoto 2012 [20]	Single-center retrospective cohort	ROBINS-I	Moderate	Small sample size, potential selection bias, and limited adjustment for confounding
Tambo 2014 [21]	Single-center retrospective cohort	ROBINS-I	Moderate	Modest sample size, potential selection bias, and incomplete control for comorbidity and baseline severity
Lu 2023 [22]	Prospective randomized clinical trial	RoB 2	Some concerns	Limited detail on allocation concealment and blinding and relatively small sample size
Mokhmalji 2001 [23]	Prospective randomized clinical trial	RoB 2	Some concerns	Open-label design, small sample size, and unclear details of outcome adjudication
Xu 2021 [24]	Prospective observational cohort	ROBINS-I	Moderate to serious	Confounding by indication (PCN often used in more severe/anatomically complex cases) and incomplete reporting of adjustment strategy
Goldsmith 2013 [25]	Retrospective single-system cohort	ROBINS-I	Moderate	Confounding by indication (PCN in sicker patients), potential selection bias, and missing data
Faw 2019 [26]	Retrospective ED cohort	ROBINS-I	Moderate	Small sample, selection bias, and limited adjustment for confounders in timing analyses

**Table 2:** Study identification, setting, population, and objective.

Study (Year)	Country	Cohort Size	Population/Setting	Primary Objective
Haas 2020 [17]	USA	311,100	National inpatient cohort: adults with UTI + obstructing stone	Assess impact of a decompression delay of ≥2 days on mortality and acute comp
Blackwell 2016 [18]	USA	10,301	State inpatient DB (FL, CA): acute nephrolithiasis with indication for decompression	Assess effect of timely ≤48 h intervention (and “weekend effect”) on mortality
Borofsky 2013 [19]	USA	1712	Nationwide Inpatient Sample: sepsis + ureteral calculi	Association of surgical decompression with mortality
Yamamoto 2012 [20]	Japan	98 (101 events)	Single-center emergency drainage for obstructive APN with calculi	Describe clinical profile and risk factors for septic shock after drainage
Tambo 2014 [21]	Japan	69	Single-center obstructive APN	Identify predictors of septic shock
Lu 2023 [22]	China	150	Prospective RCT: SIRS + ureteral stones; randomized PCN (n = 78) vs. stent (n = 72)	Compare decompression methods; identify urosepsis risk post-decompression
Mokhmalji 2001 [23]	Germany	40	Prospective RCT: hydronephrosis from stones needing diversion	Compare PCN vs. ureteral stent for urgent diversion (technical success/complications)
Xu 2021 [24]	China	110	Acute upper urinary obstruction with urosepsis; PCN vs. stent	Compare PCN vs. stent for efficacy/safety in urosepsis
Goldsmith 2013 [25]	USA	130	15 yr single-system cohort: obstructive urolithiasis with sepsis	Compare stent vs. PCN: ICU risk, LOS, failure, death
Faw 2019 [26]	USA	48	Single-center ED cohort: obstructing ureteral stone + ≥2 SIRS; all stented	Evaluate effect of stent timing (≤6–10 h vs. later) on LOS and ICU use

APN—acute pyelonephritis; ED—emergency department; ICU—intensive care unit; LOS—length of stay; PCN—percutaneous nephrostomy; RCT—randomized controlled trial; SIRS—systemic inflammatory response syndrome; USA—United States.

**Table 3:** Patient-important clinical outcomes.

Study (Year)	Mortality	Septic Shock/Severe Sepsis	ICU Use/Escalation	Length of Stay (LOS)	Other Adjusted Effects
Haas 2020 [17]	In-hospital mortality ↑ with a delay of ≥2 d (aOR, 1.29; 95% CI, 1.03–1.63)	—	—	—	AKI ↑ with delay (aOR, 1.12; 95% CI, 1.03–1.22)
Blackwell 2016 [18]	0.16% timely, ≤48 h, vs. 0.47% delayed; OR, 0.43 for timely (*p* = 0.044)	—	—	—	Weekend admission ↓ odds of timely by 26% (*p* < 0.001)
Borofsky 2013 [19]	No decompression 19.2% vs. decompression 8.82% (*p* < 0.001); lack of decompression OR, 2.6 (95% CI, 1.9–3.7)	—	—	—	National sample of 1712 sepsis + stones
Yamamoto 2012 [20]	2 deaths/98 (2.0%)	Septic shock: 21/101 events (20.8%)	Intubation: 12.9%	Median: 11 d overall; 14 d with shock vs. 10 d without (*p* = 0.008)	Shock group was older; bacteremia 71% vs. 26% (*p* < 0.001)
Tambo 2014 [21]	—	33% (23/69) septic shock	—	—	Predictors of shock: positive blood culture (59% vs. 18%; OR, 4.8), non-*E. coli* pathogen (74% vs. 33%; OR, 10.6)
Lu 2023 [22]	—	Urosepsis after decompression: 28/150 (18.7%)	—	—	Risk ↑ with pyonephrosis and higher PCT
Mokhmalji 2001 [23]	0% in-hospital deaths (reported)	—	—	—	—
Xu 2021 [24]	—	—	—	No sig. LOS difference between PCN and stent (per abstract)	Fever and WBC normalization similar between PCN and stent
Goldsmith 2013 [25]	In-hospital deaths: 0.8% overall	—	PCN ↑ ICU odds vs. stent (OR, 3.23; 95% CI, 1.24–8.41)	Longer LOS with PCN (β, 0.47; 95% CI, 0.20–0.74)	PCN cohort was sicker (APACHE II 15 vs. 11)
Faw 2019 [26]	—	—	ICU need: no difference by timing	Earlier stent cut LOS: ≤6 h 35.6 h vs. 71.6 h (*p* = 0.01); ≤10 h 45.7 h vs. 82.4 h (*p* = 0.04)	58.3% positive urine culture

aOR—adjusted odds ratio; CI—confidence interval; ICU—intensive care unit; LOS—length of stay; OR—odds ratio; PCN—percutaneous nephrostomy; PCT—procalcitonin.

**Table 4:** Summary of time-to-decompression (TTD) thresholds and associated clinical outcomes.

Study (Year)	TTD Definition/Comparison	Primary Outcome(s)	Effect Estimates
Haas 2020 [17]	A delay of ≥2 days vs. <2 days from admission to decompression	In-hospital mortality; acute kidney injury (AKI)	Mortality aOR, 1.29 (95% CI, 1.03–1.63); AKI aOR, 1.12 (95% CI, 1.03–1.22)
Blackwell 2016 [18]	Timely intervention, ≤48 h, vs. >48 h during acute stone admission	In-hospital mortality	Mortality, 0.16% vs. 0.47%; OR for timely care, 0.43 (*p* = 0.044)
Borofsky 2013 [19]	Any decompression vs. no decompression in sepsis + ureteral calculi	In-hospital mortality	Mortality, 8.82% with decompression vs. 19.2% without; OR for lack of decompression, 2.6 (95% CI, 1.9–3.7)
Faw 2019 [26]	Stent placement ≤ 6 h vs. >6 h from ED arrival	Hospital length of stay (LOS)	Mean LOS, 35.6 h vs. 71.6 h (*p* = 0.01)
Faw 2019 [26]	Stent placement ≤ 10 h vs. >10 h from ED arrival	Hospital LOS	Mean LOS, 45.7 h vs. 82.4 h (*p* = 0.04)

aOR—adjusted odds ratio; CI—confidence interval; ED—emergency department; LOS—length of stay; OR—odds ratio; TTD—time to decompression.

**Table 5:** Procedural and microbiologic outcomes.

Study (Year)	Decompression Method(s) and Technical Success	Failure/Complication	Microbiology and Labs	Timing/Anatomic Factors
Haas 2020 [17]	—	—	—	A delay of ≥2 days was associated with worse outcomes (see Table 2)
Blackwell 2016 [18]	—	—	—	Weekend admission reduced odds of timely, ≤48 h, intervention by 26%
Borofsky 2013 [19]	Decompression (stent or PCN) in 78%	—	—	Absence of decompression carried an OR of 2.6 for death
Yamamoto 2012 [20]	Stent, 89.1%; PCN, 10.9%	Intubation: 12.9%	Urine culture + 68.3%; bacteremia, 35.6%; CRP median of 16.1 mg/dL	Onset→drainage median, 3 d; stone median, 9 mm; 73.3% ureteral
Tambo 2014 [21]	Emergency drainage (mode not primary endpoint)	—	Positive blood culture was more frequent in shock (59% vs. 18%); non-*E. coli* predominant in shock (74% vs. 33%)	—
Lu 2023 [22]	Randomized: PCN, 78 vs. RUSI, 72; PCN success, 100%; RUSI, 96%	—	Urosepsis after decompression: 18.7% overall; risk ↑ with pyonephrosis/higher PCT	Definitive treatment differed between arms (*p* < 0.001)
Mokhmalji 2001 [23]	PCN success, 100% vs. stent, 80%	Major complications, 0%; PCN vs. 11% stent	—	—
Xu 2021 [24]	PCN vs. stent for urosepsis; similar clinical efficacy	Stent failure was more likely with proximal/UPJ obstruction (per abstract)	—	No significant differences in fever/WBC normalization or LOS
Goldsmith 2013 [25]	Both methods used; overall failure, 2.3% (3/130)	APACHE II was higher with PCN (15 vs. 11); stone size was larger with PCN (10 vs. 7 mm)	—	PCN → more ICU and longer LOS even after adjustment
Faw 2019 [26]	All patients stented	—	Urine culture: +58.3%	Stent ≤ 6–10 h from ED arrival reduced LOS (see Table 2)

APACHE II—Acute Physiology and Chronic Health Evaluation II; CRP—C-reactive protein; ED—emergency department; ICU—intensive care unit; LOS—length of stay; PCN—percutaneous nephrostomy; PCT—procalcitonin; UPJ—ureteropelvic junction; WBC—white blood cell count.

## Data Availability

Not applicable.

## Supplementary Materials

The following supporting information can be downloaded at: https://www.mdpi.com/article/10.3390/jcm14238546/s1, PRISMA 2020 Checklist [16].
